# Encapsulation and release of calcein from herceptin-conjugated eLiposomes

**DOI:** 10.1016/j.heliyon.2024.e27882

**Published:** 2024-03-13

**Authors:** Mah Noor Zafar, William G. Pitt, Ghaleb A. Husseini

**Affiliations:** a Biomedical Engineering Program, College of Engineering, American University of Sharjah, Sharjah, P.O. Box. 26666, United Arab Emirates; b Department of Chemical Engineering, Brigham Young University, Provo, UT, 84602, USA; c Materials Science and Engineering Ph.D. Program, College of Arts and Sciences, American University of Sharjah, Sharjah, P.O. Box. 26666, United Arab Emirates; d Department of Chemical and Biological Engineering, College of Engineering, American University of Sharjah, Sharjah, P.O. Box 26666, United Arab Emirates

**Keywords:** Emulsions, Liposomes, eLiposomes, Calcein, Drug delivery, Ultrasound, Release kinetics

## Abstract

Achieving an optimal therapeutic level is crucial in effectively eradicating cancer cells during treatment. However, conventional chemotherapy-associated systemic administration of anticancer agents leads to many side effects. To achieve the desired control over the target site, active targeting of HER2-positive breast cancer cells can be achieved by conjugating liposomal vesicles with Human Epidermal growth factor Receptor 2 (HER2) and inducing release of the encapsulated drug using ultrasound. To further enhance the delivery efficiency, nanoemulsion droplets exhibiting responsiveness to low-frequency ultrasound are encapsulated within these lipid vesicles. In this study, we prepared four different liposomal formulations, namely pegylated liposomes, emulsion liposomes (eLiposomes), HER-conjugated liposomes, and HER-conjugated eLiposomes, each loaded with calcein and subjected to a thorough characterization process. Their sizes, phospholipid concentration, and amount of antibody conjugation were compared and analyzed. Cryogenic transmission electron microscopy was used to confirm the encapsulation of nanoemulsion droplets within the liposomes. The drug-releasing performance of Herceptin-conjugated eLiposomes was found to surpass that of other liposomal formulations with a notably higher calcein release and established it as a highly effective nanocarrier. The study showcases the efficacy of calcein-loaded and Herceptin-conjugated eLiposomes, which demonstrate rapid and efficient drug release among other liposomal formulations when subjected to ultrasound. This discovery paves the way for a more targeted, efficient, and humane approach to cancer therapy.

## 1 Introduction

Current approaches to cancer treatment encompass surgery [1], radiotherapy [2,3], chemotherapy [4], immunotherapy [5], hormone therapy [6], targeted therapy [7], or a combination of these strategies [[8], [9], [10]]. Chemotherapy is one of the most widely employed methods compared to other techniques, where chemotherapeutic agents are employed to eradicate tumor cells [11]; however, conventional chemotherapy encounters challenges in achieving precise drug delivery to tumors, with approximately only 1% of the injected dose reaching the tumor site after systemic administration [12,13]. Furthermore, the heterogeneity of metastatic tumors presents varying genetic characteristics from primary tumors, making it challenging to devise a unified approach [14,15]. Breast cancer has emerged as the most prevalent cancer globally [[16], [17], [18]]. It Is the second leading cause of death globally and comprises approximately 12.5% of diagnosed cancer cases [17,19].

Surgery is deployed as the primary treatment for the removal of malignant cells. However, the choice of treatment depends on the stage, type of tumor, size, grade, proliferation rate, and involvement of lymph nodes. Surgery includes lumpectomy (partial breast tissue removal) and mastectomy (complete breast tissue removal) [20,21]. Yet surgery may not yield the most effective results for aggressive and metastatic tumors, and adjuvant therapies like chemotherapy, radiation, hormonal therapy, and targeted therapies come into play. These treatment methods enable clinicians to tailor therapeutic approaches depending on the tumor's behavior while monitoring, evaluating, and adjusting the tumor's response to chemotherapy or hormonal therapy. This helps conserve breast tissue and enhance overall patient well-being.

Roughly 25% of breast cancer cases exhibit overexpression of the Human Epidermal Growth Factor Receptor (HER2), a proto-oncogene, that correlates with malignant transformation and notably lower survival rates among breast cancer cases that have undergone lymph node metastasis. HER2 overexpression, also known as HER2-positive breast cancer, is widely used as a significant biomarker for breast cancer treatment and helps tailor personalized treatment strategies [17,22]. Monoclonal antibody (mAb)-based breast cancer treatment strategies include Trastuzumab (Herceptin) [[23], [24], [25]], Pertuzumab (Perjeta) [26,27], Margetuximab (Margenza) [28,29], Neratinib (Nerlynx) [30,31], Tucatinib (Tukysa) [[32], [33], [34]], DS-8201 (Enhertu) [35,36], and Ado-trastuzumab emtansine (Kadcyla) [37,38]. Trastuzumab (Herceptin) is a monoclonal antibody that received FDA approval in 1998 for the treatment of HER2+ breast cancers [[39], [40], [41], [42], [43]]. It specifically targets the HER2 protein overexpressed on the surface of cancer cells and blocks the cell signaling pathways for growth and division [22,44,45].

**Fig. 1:**
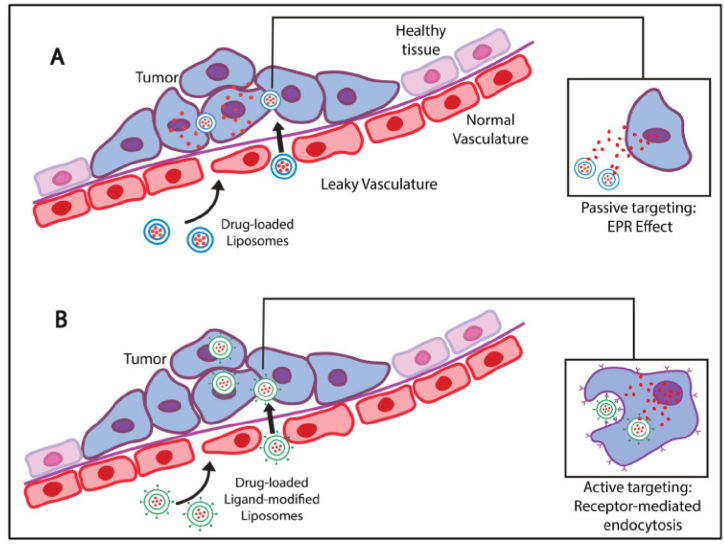
(A) Passive targeting. Liposomes <200 nm extravasate and retain at the tumor site due to its leaky vasculature and compromised lymphatic drainage system. (B) Active targeting. Liposomes accumulated at tumor site undergo receptor-mediated interactions between the targeting moiety and the overly expressed receptors on the tumor cell surfaces, enabling internalization of drug loaded liposomes into the tumor cell [58].

To address the concerns related to drug release and negative side effects of chemotherapeutic drugs, researchers are exploring the nano-sized drug delivery vehicles (<200 nm) that effectively encapsulate and transport the drug to the target tumor site [46,47]. Achieving this size is crucial to attaining passive targeting, utilizing the enhanced permeability and retention (EPR) effect for accumulation at the tumor site [[48], [49], [50]]. Additionally, active targeting is facilitated by targeting ligands on nanocarriers binding to overexpressed receptors on the cancer cell surfaces. A schematic representation of passive and active targeting mechanisms is illustrated in Fig. 1. Ayub and Wetti's study demonstrated promising outcomes by nanoparticle treatment, particularly in brain cancer [51]. Liposomes are extensively studied as carriers for imaging agents, active drugs, nucleic acids, and proteins [[52], [53], [54], [55], [56], [57]].

Liposomes, discovered as biological models by Bangham in 1961 [59], gained attention for drug encapsulation by Gregoriadis in 1973 [60]. Resembling cell membranes in composition, liposomes are biocompatible, biodegradable, non-toxic, stable in the physiological environment, and reduce the toxicity of the encapsulated drug [[61], [62], [63], [64]]. Many widely used liposomal nano-drug delivery systems with encapsulated hydrophobic and hydrophilic drugs, adapt to the tumor environment based on pathophysiology [61,[65], [66], [67], [68]]. These systems offer a controlled drug distribution and release at the target site, reducing the need for frequent dosing, potentially improving patient compliance, and minimizing some side effects [66,[69], [70], [71]].

To mitigate the rapid clearance of liposomes by the reticuloendothelial system (RES), liposomes are coated with flexible polyethylene glycol (PEG) polymer [72]. PEG-coated liposomes experience reduced immune system recognition, enhanced stability in vivo, and prolonged circulation, allowing sustained presence in the bloodstream for targeted drug delivery [[73], [74], [75]]. Furthermore, adding targeting moieties to liposomal surfaces enhances tumor targeting capabilities while minimizing off-target effects and interaction with healthy cells and minimizing the off-target effects [22,46,[76], [77], [78], [79], [80]]. The personalized therapeutic approach exemplified by Herceptin-conjugated liposomes for breast cancer therapy, shows significant improvements in anticancer drug uptake and cellular toxicity levels [78,81].

**Fig. 2:**
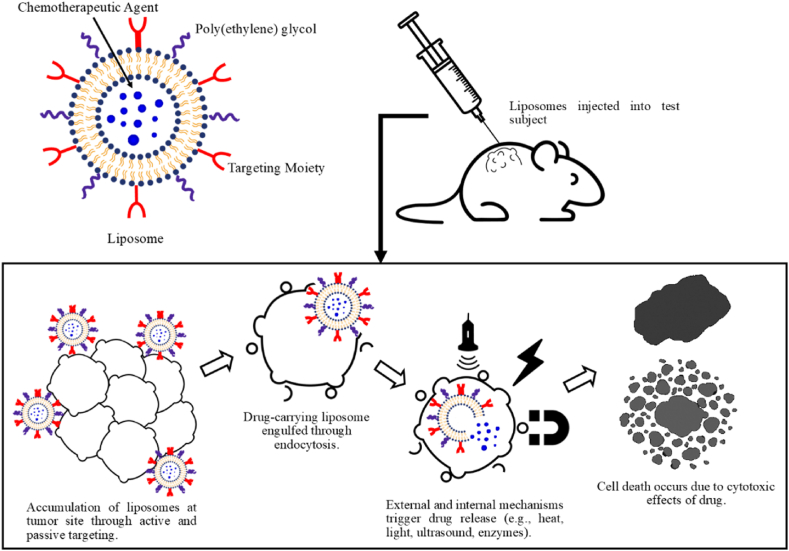
A visual representation of a drug delivery system based on smart liposomes to treat solid tumors. Adapted from Ref. [58].

Recent studies emphasize smart drug delivery systems (SDDS) for targeted and strategic drug delivery, specifically nanoparticles releasing at the target microenvironment. Smart liposomes respond to internal triggers like pH levels [82], enzyme activity [83], redox gradients [84,85], and hormone levels [86] as well external stimuli such as temperature [87,88], magnetic field [89], ultrasound (US) [90], and high-energy radiation [22,[91], [92], [93]]. These systems enable personalized and targeted medicine by accurately controlling the release of therapeutic agents according to the patient's condition [47,79]. The choice of using low-frequency ultrasound (LFUS) in this study is due to its safe, non-invasive nature, customizable parameters, and precise targeting capabilities [90,[94], [95], [96], [97]]. Fig. 2 Represents a schematic illustration of a smart liposome-based drug delivery system.

Crucial ultrasound parameters include frequency, power density, and pulse duration. Drug delivery systems employ high-intensity and low-intensity focused ultrasound to induce synergistic effects in the controlled release of chemotherapeutic drugs. Thus, it is advantageous to use pulsed-wave (PW) Doppler US to allow dissipation of heat between successive pulses [[98], [99], [100], [101]]. Exposure to high-frequency ultrasound locally heats the body tissues; this helps the accumulation of nanoparticles at the target site and activates temperature-sensitive nanoparticles. However, high hyperthermia >43 °C ceases tissue blood flow, leading to rapid cell death (necrosis).

**Fig. 3:**
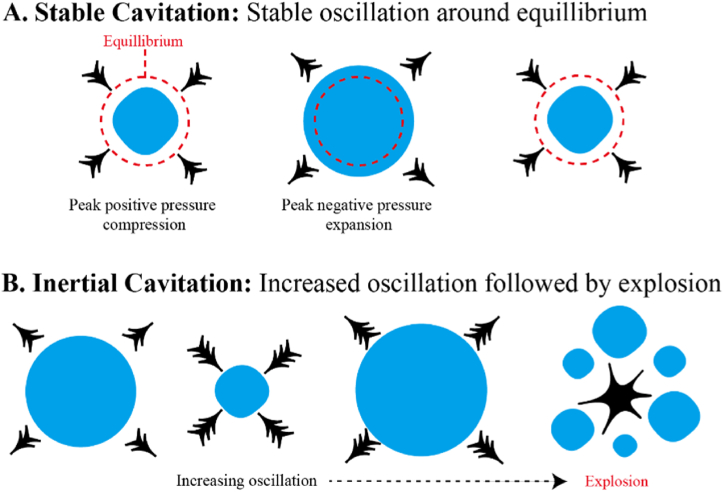
Ultrasound-induced thermal effects on tissues.

**Fig. 4:**
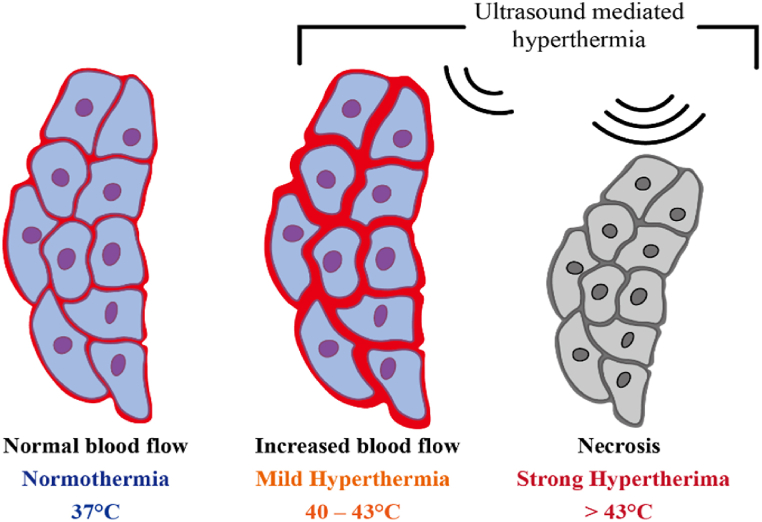
Mechanical effects of ultrasound upon microbubbles producing stable cavitation or inertial cavitation.

**Fig. 5:**
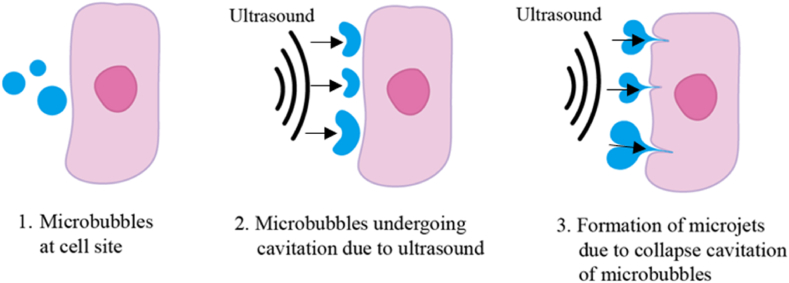
Schematic illustration of micro jets created by microbubbles as a result of collapse cavitation.

Fig. 3 Illustrates a visual representation of the thermal effects induced on tissues upon exposure to ultrasound. Moreover, the US propagates through a medium as high-pressure or low-pressure waves, inducing pressure variations within a medium. This imparts energy to particles of the propagating medium and leads to the production of small gas pockets. This phenomenon is called acoustic cavitation. Variations in pressure cause gas bubbles to linearly oscillate, creating strong shear forces that temporarily permeabilize cell membranes and help penetrate the nanocarriers into the tumor tissue. Fig. 4 Depicts a schematic representation of the mechanical effects of ultrasound, with microbubbles (MBs) undergoing stable cavitation or inertial cavitation. Moreover, when oscillations become non-linear with the increase in US intensity, rapid growth and subsequent implosion of gas bubbles take place; this is called collapse cavitation. The implosion is accompanied by high-pressure shock waves and sometimes the production of a sonic jet that leads to the sonoporation of the cell membrane [[101], [102], [103], [104], [105]]. Fig. 5 Depicts an illustration of microjets created by microbubbles undergoing collapse cavitation.

Salkho et al. studied the potential of an endogenous ligand, i.e., estrone, and investigated the release of doxorubicin from estrone-conjugated liposomes triggered by ultrasound waves at different frequencies and power densities. The study confirms significantly higher drug uptake in Estrogen receptor (ER)-positive (MCF-7) cell lines compared to ER-negative (MDA-MB-231) cell lines. The application of low-frequency ultrasound further enhanced the uptake, promising a non-immunogenic and site-specific biomedical approach for ER-positive breast cancer therapy [106]. Awad et al. investigated the liposomal conjugation of human serum albumin (HSA) for the delivery of calcein to breast cancer cells. The results reveal significantly higher calcein uptake by breast cancer cell lines (MDA-MB-231 and MCF-7) with HSA-PEG liposomes compared to non-targeted control liposomes. Additionally, exposure to low-frequency ultrasound (LFUS) significantly enhances calcein uptake, indicating the potential of combining targeted liposomes formulations with ultrasound for improved drug delivery to breast cancer cells [107]. Elamir et al. studied the cellular toxicity of calcein and Doxorubicin-loaded Trastuzumab-conjugated liposomes in the HER2-positive cell line SKBR3. The study confirmed an increased drug uptake and higher cellular toxicity exhibited by immunoliposomes compared to control liposomes. Furthermore, sonication with LFUS further improved drug uptake, potentially enhancing efficiency and reducing the cytotoxicity associated with antineoplastic drugs [25].

Despite their clinical use, liposomes still encounter limitations in swiftly achieving optimal chemotherapeutic drug concentrations at the target site, due to their adequately stable liposomal membranes and inherent lack of responsiveness to ultrasound. This reduces their potential effectiveness against cancer. Researchers have investigated strategies to enhance the responsiveness of liposomes to ultrasound by incorporating MBs, nanobubbles, and phase-changing nanoemulsion within or upon droplets [75,[108], [109], [110], [111], [112], [113], [114]]. Microbubbles are composed of lipid shells filled with perfluorocarbon gas. Olsman et al. investigated the effect of focused ultrasound (FUS) and MBs on the transferrin (Tf) targeted liposomes in enhancing the permeability of the blood-brain barrier (BBB) in rats, which overexpress Tf receptors in the BBB. The study revealed that FUS and microbubbles helped safely increase blood-brain barrier permeability and recorded a 40% increase in the accumulation of Tf-targeted liposomes in the brain hemisphere compared with isotype immunoglobulin G (IgG) liposomes [115].

However, the size of MBs, (diameter greater than about 1 μm) limits transport within the tumor vasculature and precludes MBs from benefiting from the EPR effect. Nevertheless, MBs have been employed as intra-vascular agents to actively target endothelial markers such as VEGFR2 and αvβ3 integrin [116,117]. The large size of microbubbles (Fig. 5) incentivized the development of nano-scale-sized nanobubbles and nanoemulsions that would easily extravasate into the tumor tissues and become endocytosed into the tumor cells. Upon exposure to ultrasound, nanoemulsion droplets of perfluorocarbon liquids transform from liquid to gas, resulting in an increased volume within the liposomal vesicle, subsequently leading to rupture and prompt release of the enclosed drug [58,114,[118], [119], [120], [121], [122]].

**Fig. 6:**
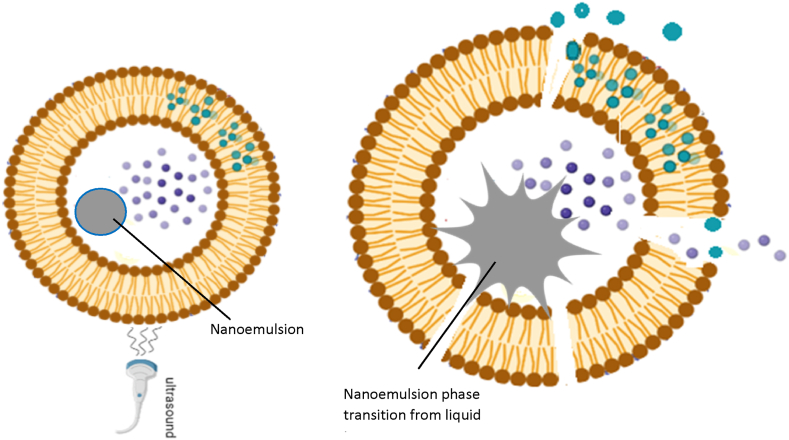
Schematic illustration of drug release from an eLiposome before and after exposure to ultrasound. Adapted from [58].

This is known as acoustic droplet vaporization (ADV). It is important to note that lipid bilayers can tolerate only a 3% increase in volume before reaching the rupture point. This substantial increase in volume upon phase change is sufficient to rupture both eLiposomes and the endosome [[122], [123], [124]]. This phenomenon helps attain the desirable therapeutic dose while regulating and controlling the release of anticancer drugs at the target site. Fig. 6 Provides a visual representation of drug release from eLiposomes upon exposure to ultrasound.

Lattin and Pitt designed experiments to investigate the performance of eLiposomes and liposomes at physiological temperatures (37 °C). These experiments revealed the stability and capability of eLiposomes to sequester drugs at physiological temperatures. Experiments employed a fluorometer that measured fluorescence in a heated water bath at incubation times of 3, 10, 20, and 30 min. They repeated the process for both eLiposomes with large (450 nm) and (100 nm) emulsions. No calcein release was observed from the samples mentioned above, signifying that heating to body temperature alone cannot render eLiposomes unstable. Finally, Triton-X was used to lyse the eLiposomes, which released all calcein sequestered in the eLiposomes, thus indicating that eLiposomes are very stable at physiological temperatures. They further compared the ultrasound-induced release of the encapsulated model drug, calcein, from eLiposomes (containing PFC_5_ and PFC_6_) with the two negative controls (without the droplets and with droplets outside the liposomes vesicle). The eLiposomes showed significantly higher calcein release than both control groups, which was attributed to the emulsion droplets inside the liposome vesicles disrupting its membrane structure from within the eLiposomes and releasing calcein.

**Fig. 7:**
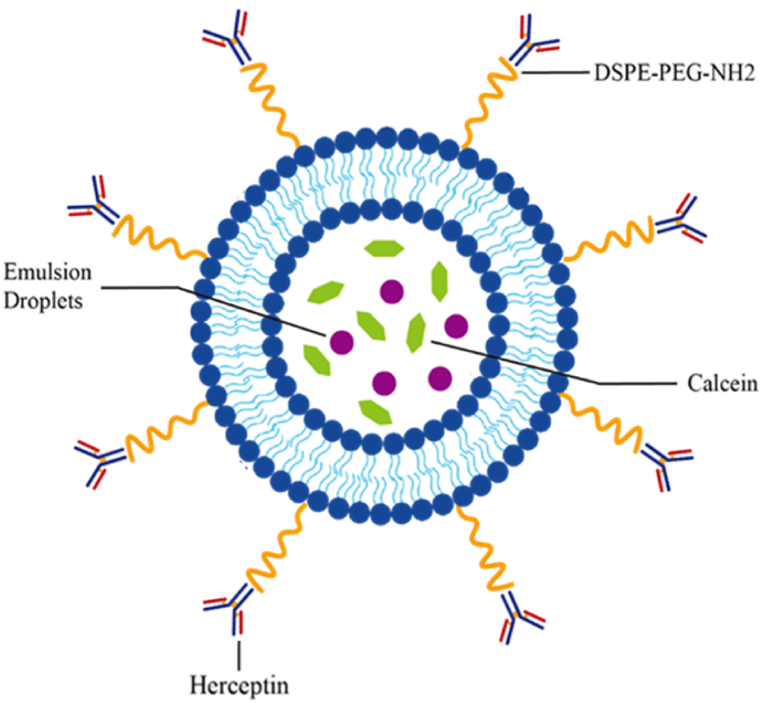
Structural illustration of a Herceptin (Trastuzumab)-conjugated eLiposome.

The eLiposomes showed 3–4 times more calcein release than the control groups, which increased further upon increasing ultrasound power intensity and time of exposure. However, after a certain amount of time or energy, no further increase was observed upon increasing the exposure. Their study also reported that an increase in power density resulted in an increased tissue temperature; however, this increase in temperature was not responsible for the significantly higher release from eLiposomes compared to conventional liposomes. Furthermore, they studied the behavior of PFC_5_ eLiposomes and control (conventional) liposomes as a function of US frequency (varying from 20 kHz to 525 kHz) and mechanical indices (MI = 0.53 at 5 W/cm^2^ and MI = 1.41 corresponding to 35 W/cm^2^). In this study, PFC_5_ eLiposomes and control liposomes were exposed to PW ultrasound for 2–30 s with 525 kHz at 20 kHz pulse repetition frequency. Their study demonstrated that frequency significantly affects the phase transition of emulsion droplets. They concluded that lower frequency offers a long window of negative pressure, allowing more time for bubble nucleation and gas expansion; thus, it was concluded that increasing the frequency decreases the threshold of acoustic vaporization. PFC_5_ eLiposomes showed a significant difference in their drug release compared to control liposomes: about 2–3 times and 3–5 times more drug release was demonstrated by PFC_5_ eLiposomes when exposed to 5 W/cm^2^ and 35 W/cm^2^, respectively; however, the study showed no significant release from control liposomes with the changes in intensities [113]. In the present study, pegylated liposomes encapsulating calcein and nanoemulsion droplets were formulated and functionalized with the monoclonal antibody Herceptin (see Fig. 7). The release of calcein from conventional liposomes, eLiposomes, HER-conjugated liposomes, and HER-conjugated eLiposomes was catalyzed by employing low-frequency ultrasound as a trigger. This is the first study where targeted eLiposomes with an antibody are used to demonstrate triggered drug release. eLiposomes are used in this study because they are more echogenic and require less ultrasound power to destroy and release their contents at the targeted site.

## 2 Materials and Methods

### 2.1 Materials

The liposomes were formulated using specific phospholipids: dipalmitoylphosphatidylcholine (DPPC) and 1,2-distearoyl-*sn*-glycero-3-phosphoethanolamine-N [amino (polyethylene glycol)-2000] (DSPE-PEG (2000)-NH_2_) are obtained from Avanti Polar Lipids Inc. (Alabaster, AL, USA.). Calcein disodium salt (C_30_H_24_N_2_Na_2_O_13_), cholesterol, bicinchoninic acid kit, Sephadex G-100, Triton X-100, ammonium ferrothiocyanate (AF) are acquired from Sigma Aldrich (St. Louis, Missouri, USA). Trastuzumab (Herceptin) was purchased from Hoffmann-La Roche Limited (Basel, Switzerland, supplied by Aster Pharmacy, Sharjah, UAE). The 0.2-μm and 0.05-μm polycarbonate membrane filters and filter support were purchased from Whatman PLC (Maidstone, England, U.K.). 2,4,6 trichloro-1,3,5 triazine (cyanuric chloride) was procured from Sigma-Aldrich (St. Louis, MO, US). Perfluoropentane (PFC_5_) was acquired from Strem Chemicals (Newburyport, MA, USA).

### 2.2 Preparation and characteristics

#### 2.2.1 Preparation of PFC_5_ nanoemulsion droplets

**Fig. 8:**
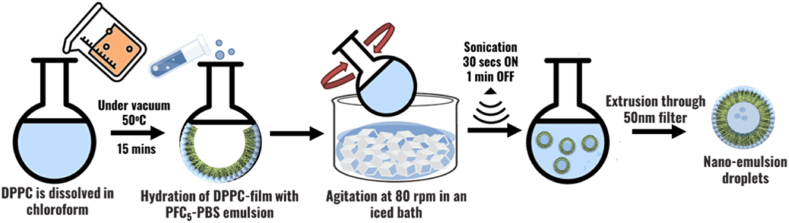
Comprehensive schematic representation illustrating the experimental setup and steps involved in the preparation of nanoemulsion droplets.

Emulsion droplets were prepared by dissolving 10 mg of DPPC in 1 mL of chloroform. The solution was evaporated onto the surface of a round-bottomed flask at 50 °C for 15 min. The procedure was conducted under a vacuum using a rotary evaporator (rotovap). The dried film was hydrated by introducing 1.2 mL of PBS (phosphate-buffered saline). 0.6 mL of PFC_5_ (perfluoro pentane) was added to the solution. The assembly was rotated on an iced bath for 20 min and subsequently sonicated through pulses of 30 s in an active state, followed by a 1-min pause interval between each sonication cycle. Sonication was done using a 40-kHz sonicator bath (Elma D-78224, Melrose Park, IL, USA). The size of nanoemulsion droplets was reduced by extruding through polycarbonate membrane filters with a pore size of 0.05 μm (Hamilton, Reno, NV) [109,125]. Fig. 8 Provides a schematic representation of the experimental setup and preparation for nanoemulsion droplets.

#### 2.2.2 Preparation of calcein-encapsulated DSPE-PEG-NH_2_ control liposomes

**Fig. 9:**
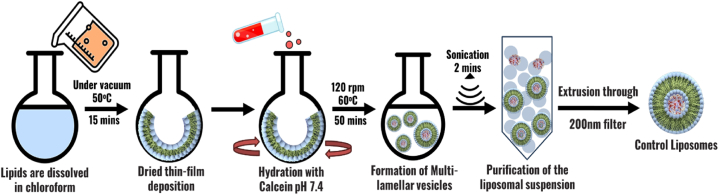
Comprehensive schematic representation illustrating the experimental setup and steps involved in the preparation of control liposomes.

The preparation of liposomes was carried out using the thin-film hydration method. The formulation was composed of cholesterol, DPPC, and DSPE-PEG (2000)-NH_2_ in molar ratios of 30:65:5, respectively, which was dissolved in 4 mL of chloroform within a round-bottomed flask. Subsequently, the chloroform was evaporated via a rotary evaporator under a vacuum at a temperature of 50 °C for 15 min, leaving behind a thin film on the inner surface of the flask. The lipid film was subjected to hydration using 2 mL of a 50-mM calcein solution with a carefully adjusted pH of 7.4. The assembly was agitated at 120 rpm at 60 °C for 50 min. Unilamellar liposomal vesicles were produced by sonicating the assembly for 2 min, in a 40-kHz sonicator bath (The Elma D-78224 system from Melrose Park, IL, USA) [25]. The liposomes were extruded through a polycarbonate Nuclepore Whatman filter with a pore size of 200-nm acquired from Avanti Polar Lipids, Inc. (Alabaster, AL, USA). The extruded liposomes were purified by gel extrusion chromatography using a Sephadex G-100 column to obtain the uniform-sized distribution of liposomes free from calcein [126,127]. A schematic representation of the experimental setup and preparation for control liposomes is provided in Fig. 9.

#### 2.2.3 Preparation of (emulsion liposomes) eLiposomes

**Fig. 10:**
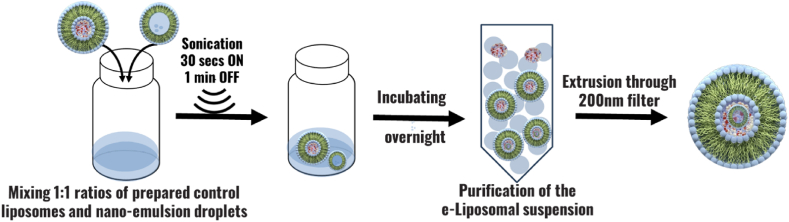
Comprehensive schematic representation illustrating the experimental setup and steps involved in the preparation of eLiposomes.

In this technique, eLiposomes were prepared by adding previously synthesized nanoemulsion droplets and calcein-encapsulated control liposomes at an equivalent amount of (0.5 mL) maintaining a volume ratio of 1:1. The mixture was subjected to sonication while sitting within an ice bath, to keep PFC_5_ from evaporating. The sonication cycle consisted of pulses of 10 s of active sonication and 60 s of no sonication, and this sequence was repeated three times. The eLiposomes were extruded through a polycarbonate Nuclepore Whatman filter with a pore size of 50-nm acquired from Avanti Polar Lipids, Inc. (Alabaster, AL, USA) and purified by gel extrusion chromatography using a Sephadex G-100 column to obtain the uniform-sized distribution of eLiposomes free from calcein and empty liposomes [122]. Fig. 10. Provides a schematic illustration of the experimental setup and preparation for eLiposomes.

#### 2.2.4 Preparation of trastuzumab-conjugated liposomes

**Fig. 11:**
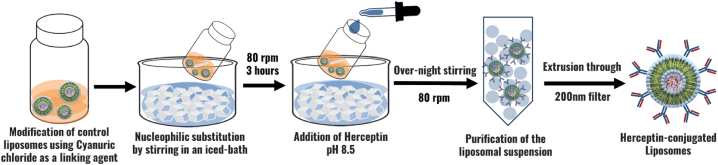
Comprehensive schematic representation illustrating the experimental setup and steps involved in the preparation of Herceptin-conjugate liposome.

**Fig. 12:**
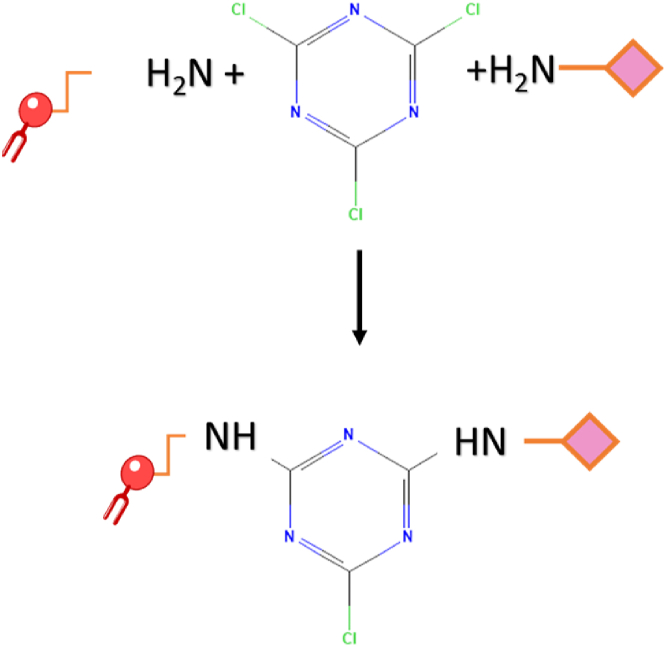
The conjugation of Herceptin to the DSPE-PEG-NH_2_ chains of our control liposomes with cyanuric chloride as a coupling agent.

The strategy of conjugating herceptin to the DSPE-PEG-NH_2_ chains of our control liposomes with cyanuric chloride as a coupling agent is shown in Fig. 12. Initially, control liposomes were modified by utilizing 2,4,6 trichloro-1,3,5 triazine (cyanuric chloride) as a coupling agent to initiate the conjugation process. First, cyanuric chloride was dissolved into acetone, yielding a 10 mg/mL solution. Next, 9.23 μL of cyanuric chloride was diluted with de-ionized water to avoid potential disruption of liposomes. The resulting solution was combined with liposomes (1 mL) in a vial. The nucleophilic substitution of the proton on the –NH_2_ group located on the surface of liposomes with chloride sites on cyanuric chloride was carried out within an iced bath by stirring at 80 rpm for 3 h. The next step involved dissolving Trastuzumab in 0.5 mL of borate buffer with a pH of approximately 8.5. Following 3-h incubation. Trastuzumab solution was introduced into the modified liposome assembly with stirring sustained at 80 rpm overnight. Sephadex G-100 column was prepared to enable the purification of liposomes. The resultant purified solution was stored at 4 °C [25,127,128]. Fig. 11, Fig. 12. Provide a schematic illustration of the experimental setup and preparation for Herceptin-conjugated liposomes and the conjugation of the Herceptin to the DSPE-PEG-NH_2_ chains of the control liposomes, respectively.

#### 2.2.5 Preparation of trastuzumab-conjugated eLiposomes

**Fig. 13:**
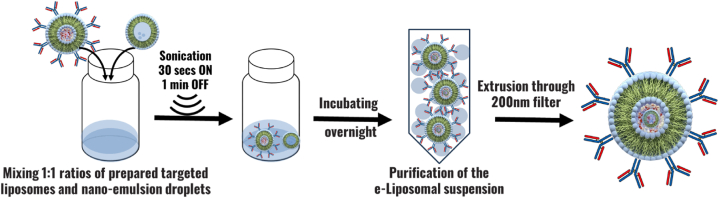
Comprehensive schematic representation illustrating the experimental setup and steps involved in the preparation Herceptin-conjugated eLiposomes.

To prepare Trastuzumab-conjugated eLiposomes, previously prepared nanoemulsion droplets (0.5 mL) were introduced into (0.5 mL) Trastuzumab-conjugated liposomes to establish a 1:1 volumetric ratio. The mixture was subjected to three sonication cycles of 10 s followed by 60 s of no sonication. This facilitates the encapsulation of nanoemulsion droplets within Trastuzumab-conjugated liposomes. Gel exclusion chromatography using a Sephadex G-100 column was employed to purify the Trastuzumab eLiposomes from excess Trastuzumab and empty liposomes. Fig. 13. Provides a schematic illustration of experimental setup and preparation for Herceptin-conjugated eLiposomes.

#### 2.2.6 Size and polydispersity evaluation using Dynamic Light Scattering (DLS)

Dynamic Light Scattering (DLS) was employed (Dynapro® Nanostar™ provided by Wyatt Technology Corp., Santa Barbara, CA, USA) to evaluate the average dimensions and polydispersity index (PDI) of both liposomes and eLiposomes. This evaluation was aimed to ensure that the liposomal formulations did not exceed the 200 nm diameter range, thus facilitating the enhanced permeability and retention effect (EPR) [129]. The random motion of particles immersed in a liquid medium at a temperature of 25 °C, also called Brownian motion, determines the hydrodynamic radii and variability in particle size. The particles’ rate of Brownian motion or translational diffusion coefficient (D), can be converted to particle size using the Stokes-Einstein equation [130]:

(1) $$D=\frac{k_{B}T}{6\pi \eta R_{H}\;}$$

where *D* = translational diffusion coefficient (m^2^/s), *k*_*B =*_ Boltzmann constant (m^2.^ kg/K/s^2^), *T =* solution temperature (K), *η =* viscosity (Pa^.^s), and *R*_*H*_ = hydrodynamic radius (m).

#### 2.2.7 Quantification of the lipid content of the prepared liposomal formulations using Stewart assay

The phospholipid content of liposomes was estimated through the Stewart assay. This method forms the distinctive complex between phospholipids and ammonium ferrothiocyanate (FTC), whose maximal absorbance is 485 nm. The prepared liposome samples (100 μL) were vacuum-dried in a round-bottomed flask. The dried liposome film was dissolved with chloroform (1 mL) and subjected to sonication for 10 min to break the liposomes into their constituent lipids. The liposomes solution obtained was transferred into a centrifuge tube along with 2 mL of ammonium ferrothiocyanate and centrifuged at 1000 rpm for 10 min, resulting in a biphasic distribution with an upper dark layer and a bottom transparent chloroform layer. The upper layer was discarded, whereas the bottom transparent layer was transferred into a quartz cuvette. Its optical density was measured using Evolution™ 60 S ultraviolet–visible (UV–Vis) spectroscopy (ThermoFisher Scientific, Madison, WI, USA) along with the VISIONlite software at a max absorbance peak of 485 nm with chloroform used as a reference blank with an optical density of zero. Unlike other assays, the presence of inorganic phosphate does not impact the accurate measurements of the phospholipids. A total of 8 recordings were taken, with two serving as blanks. The procedure was repeated for targeted liposomes.

#### 2.2.8 Quantification of antibody conjugation using bicinchoninic acid (BCA) assay

The Bicinchoninic acid (BCA) assay assessed the Trastuzumab conjugation efficiency to liposomes. It is a colorimetric detection method using a highly sensitive chelating agent to quantify protein concentrations within a sample. The bicinchoninic acid reagent was prepared by mixing 4.5 mL of QuantiPro™ buffer QA, 4.5 mL of QuantiPro™ buffer QB, and 180 μL of CuSO_4_ solution using the QuantiPro™ BCA kit purchased from Sigma-Aldrich Chemie GmbH (supplied through LABCO LLC., Dubai, UAE). BCA assay relies on the proteins’ ability to reduce cupric ions Cu^+2^ to cuprous ions Cu^+1^ in an alkaline environment, called the biuret reaction. The chelation of two bicinchoninic acid molecules with one cuprous ion forms the purple-colored complex. Eight microfuge tubes were prepared, containing varying volumes of liposomes, PBS, and BCA reagent, including two blank samples comprising solely PBS and BCA reagent, to establish a baseline for precise comparison. All tubes were prepared to achieve a final volume of 2 mL. The tubes were placed in a water bath with a temperature of 60 °C. After incubation, samples were allowed to cool down to room temperature and transferred into cuvettes to quantify maximal absorbance at a specific wavelength of 562 nm. The Evolution™ 60 S ultraviolet–visible (UV–Vis) spectroscopy (ThermoFisher Scientific, Madison, WI, USA) was employed for the assessment, streamlined through utilizing VISIONlite software. Moreover, the Stewart assay results, combined with the molecular weight of Trastuzumab and DPPC, were used to quantify the number of trastuzumab molecules per liposomal vesicle.

#### 2.2.9 Cryogenic transmission electron microscopy (Cryo-TEM)

Cryo-electron microscopy was employed to visualize the unaltered physical form of eLiposomes in their native states. Cryo-TEM aimed to validate the successful encapsulation of nanoemulsions within the liposomes. The samples were rapidly frozen using liquid nitrogen to achieve extremely low temperatures below −150 °C to maintain structural integrity and allow electron microscopy imaging in a nearly native state. Visualization was done using a TEM instrument, enabling the detailed examination of the eLiposomes’ inner structure.

### 2.3 Low-frequency ultrasound release of calcein

Calcein is a fluorescent molecule characterized by an excitation wavelength of 495 nm and an emission wavelength of 515 nm. At low concentrations, the emission is proportional to the molar concentrations, but at sufficiently high concentrations, there is total self-quenching (no emission). Thus, liposomes containing high calcein concentrations have no fluorescence, but when calcein is released from the liposomes, the fluorescence is proportional to the amount released. To initiate the release of concentrated calcein from both liposomal and eLiposomal samples, low-frequency ultrasound (LFUS) at 20 kHz was applied using an ultrasonic probe (model VCX750, Sonics & Materials Inc., Newtown, CT, USA), and the changes in fluorescence emission were monitored using a QuantaMaster QM 30 Phosphorescence Spectrofluorometer (Photon Technology International, Edison NJ, USA).

The sample under test was prepared by diluting 75 μL of liposomes in 3 mL of PBS with a pH of 7.4 within a fluorescence cuvette. The slits of the spectrofluorometer sample compartments were set to 1.25 mm each. The sample cuvette was placed inside the spectrofluorometer chamber with an ultrasonic probe inserted approximately 2 mm into the fluid in the cuvette through an opening in the instrument chamber. The experiment was conducted at room temperature, commencing with establishing and recording the initial baseline fluorescence for 50 s without sonication, followed by a series of pulsed ultrasounds (US) with cycles of 20 s of sonication followed by 20 s of no ultrasound, repeated for 7 min.

The calcein release was monitored at three different ultrasonic power settings: 20%, 25%, and 30%, corresponding to power densities of 6.2, 9.0, and 10.0 mW/cm^2^, respectively, as measured by the hydrophone. The US sonication cycles were repeated for 7 min or until a plateau was observed; at this point, 50 μL of Triton X-100 (Tx100) was delivered into the cuvette to lyse the liposomes and release all the encapsulated calcein.

The cumulative fraction of calcein released (CFR) from liposomes was calculated using the following equation [131]:

(2) $$CFR=\frac{F_{US}-F_{i}}{F_{tot}-F_{i}}*100$$

In this equation, *F*_*i*_ is the baseline intensity, *F*_*US*_ is the intensity at US application time (20 kHz), and *F*_*tot*_ is the maximum fluorescence obtained after lysing all liposomes with Tx100.

### 2.4 Kinetic modeling of drug release

Mathematical models are essential in drug delivery systems, enabling the monitoring, assessing, and optimizing of drug release kinetics. It is vital to understand, model and control the release of the encapsulated drugs to achieve the desired therapeutic outcome. Drug release is dependent upon diffusion across the membrane, leakage through pores in the membrane, and mechanical rupture of the liposome membrane, all of which are related to the properties of the liposomes, its composition, the type of encapsulated drug, and the conditions of release [128,132]. Employing mathematical models to fit data provides an understanding of the liposomal release process and aids in designing effective smart liposomes. Two distinct mathematical models, zero-order and first-order, were employed to evaluate the acoustic release kinetics of calcein associated with the release process. These models helped quantify physical parameters linked to drug release, such as the membrane integrity and the drug's diffusion coefficient, thus providing insights into the delivery processes that can control drug distribution within the system.

#### 2.4.1 Zero-order kinetics modeling

The zero-order model exhibits a consistent rate of change in the amount of the drug released over a specific interval. The concentration of the free drug present in a solution at a particular time can be denoted by *C*_*t*_. According to the underlying hypothesis, the following equation [133] can represent the behavior of the drug concentration in the solution:

(3) $$\frac{dC_{t}}{dt}=k_{0}$$

where *k*_*0*_ is a constant representing the rate at which the drug is released, regardless of the concentration. Integrating the above differential equation within the time bounds of 0 to a given time (*t*) shows that for zero-order kinetics, the concentration of the released drug will increase linearly with time.

#### 2.4.2 First-order kinetics modeling

In a first-order model of drug release, the amount of calcein released is proportional to the amount still sequestered at high concentrations inside liposomes. As calcein is released, the sequestered amount decreases, which decreases the rate of release. If $C_{free}\left(t\right)$ represents the amount released at any time *t*, then $\left(C_{0}-C_{free}\left(t\right)\right)$ represents the amount still sequestered, where $C_{0}$ is the initial concentration of all sequestered calcein. The following equation [134] presents the differential equation showing that the rate of release is proportional to what remains in the liposomes, with proportionality constant *k*, also called a rate constant:

(4) $$\frac{dC_{free}\left(t\right)}{d\left(t\right)}=k\left(C_{0}-C_{free}\left(t\right)\right)$$

Integration of this equation within the time bounds of 0 to a given time (*t*) produces a plot of released calcein that initially climbs quickly by constantly slowing its climb but never exceeds the release of all calcein, and is given by

(5) $$C_{free}\left(t\right)=C_{0}\left(1-e^{-kt}\right)$$

## 3 Results and discussion

### 3.1 Characterization of liposomes

#### 3.1.1 Estimation of size using dynamic light scattering (DLS)

**Fig. 14:**
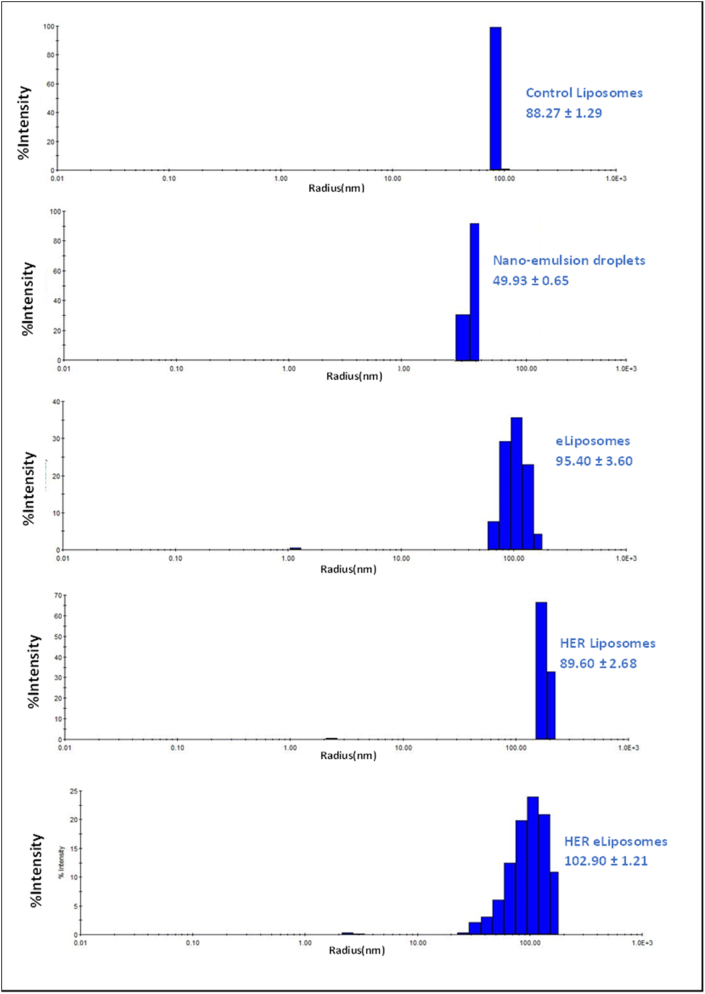
Size distribution of control liposomes, nanoemulsions, eLiposomes, HER-liposomes and HER-eLiposomes.

Dynamic light scattering was employed to evaluate the size and polydispersity index (PDI) of the three batches of each sample type: control liposomes, nanoemulsions, eLiposomes, and targeted liposomes and eLiposomes. As stated earlier, measuring the radii evaluated the possibility of leveraging the enhanced permeability and retention (EPR) effect with these particles. Fig. 14 depicts that the size of the nanocarriers falls within the EPR range (10–200 nm in diameter). Furthermore, the polydispersity index (PDI) was also evaluated to ascertain the uniformity of the size of all liposomal formulations within each sample. A PDI of up to 20% or less is suitable for drug delivery applications [129]. Table 1, Table 2 present the average diameters and percentage polydispersity index (%Pd) values.

**Table 1:** DLS results for control and HER-conjugated liposomes.

	Control Liposomes		HER Liposomes	
Batches	Radius (nm)	PDI (%Pd)	Radius (nm)	PDI (%Pd)
Batch 1	89.70	10.40	91.50	11.20
Batch 2	87.90	13.60	86.80	14.20
Batch 3	87.20	12.00	90.50	16.20
**Average**	**88.27 ± 1.29**	**12 ± 1.60**	**89.60 ± 2.48**	**13.87 ± 2.52**

**Table 2:** DLS results for emulsions, eLiposomes, and HER-conjugated eLiposomes.

	Emulsions		eLiposomes		HER eLiposomes	
Batches	Radius (nm)	PDI (%Pd)	Radius (nm)	PDI (%Pd)	Radius (nm)	PDI (%Pd)
Batch 1	50.60	16.00	99.00	20.60	104.20	29.10
Batch 2	49.90	12.50	95.40	16.40	102.70	26.10
Batch 3	49.30	9.20	91.80	14.50	101.80	24.40
**Average**	**49.93 ± 0.65**	**12.57 ± 3.40**	**95.40 ± 3.60**	**17.17 ± 3.12**	**102.90 ± 1.21**	**28.20 ± 1.82**

Statistical analysis was conducted on liposomal formulations that revealed that Herceptin-conjugated liposomes exhibited a slightly larger size than control liposomes, with a statistically significant difference (*p*-value = 0.022). This can be attributed to the attachment of some Herceptin molecules (molecular weight of 100 kDa) to the liposomal surface, contributing to an estimated 2.5 nm increase in liposomal dimensions.

Comparatively, the radius of eLiposomes and control liposomes exhibited a statistically significant difference (*p*-value = 0.0339), whereas the difference between eLiposomes and HER liposomes did not attain statistical significance (*p*-value = 0.119). This discrepancy can be potentially attributed to the encapsulation of 50 nm nanoemulsions within the liposomal structure. It is noteworthy that eLiposomes lacked Herceptin moiety on their surface. The size distribution of eLiposomes is provided in Table 2.

Considering the previously mentioned observations, it is notable that Herceptin-conjugated eLiposomes exhibited the largest size among all liposomal formulations. This can be attributed to the coexistence of encapsulated nanoemulsions and the Herceptin moieties attached to the liposomal surface. However, it is pertinent to highlight that all the samples demonstrated size within the 200 nm range, enabling the potential exploitation of the EPR effect with these particles.

#### 3.1.2 Quantification of total lipid concentration using the Stewart assay

In this study, all liposomal formulations, including emulsions, were prepared using DPPC as the primary lipid component, characterized by its light absorption at 485 nm. To determine the accurate lipid content, a calibration curve was established using known DPPC concentrations in mg/mL against the corresponding absorbed wavelength (see Materials and Methods section). Table 3 provides the results for DPPC content within control liposomes and HER-conjugated liposomes.

**Table 3:** Stewart Assay results for the amount of lipid in control liposomes and HER-conjugated liposomes.

Batches	Lipid in control Liposomes (mg/mL)	Lipid in HER Liposomes (mg/mL)	Control-to-HER liposome ratio (mg/mg)
Batch 1	14.97	6.13	2.44
Batch 2	14.66	6.74	2.17
Batch 3	10.12	6.05	1.67
**Average**	**13.25 ± 2.71**	**6.31 ± 0.38**	

The observations from Table 3 highlight approximately twice as much lipid content in control liposomes when compared with HER-conjugated liposomes. This trend can be attributed to the additional liposomal column purification step to enhance targeted liposome quality following Herceptin conjugation. However, it results in the entrapment of lipids within the porous structure of the column beads, ultimately decreasing the final lipid concentration.

#### 3.1.3 Estimation of protein content through BCA assay

**Fig. 15:**
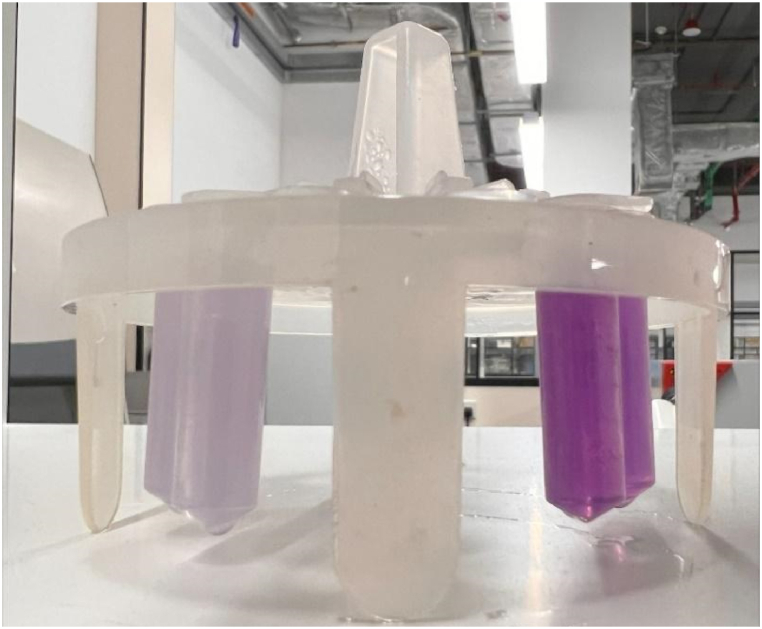
The difference in color intensity observed between control liposomes (left) and Herceptin-conjugated liposomes (right).

The BCA results showed a difference in color intensity between the control and HER-conjugated liposomes, as illustrated in Fig. 15. Control liposomes exhibit a subtle purple hue, which can be attributed to the presence of amine groups in the DSPE-PEG-NH_2_ compound. In contrast, immunoliposomes demonstrate a vivid purple color owing to the simultaneous presence of Trastuzumab along with the DSPE-PEG-NH_2_. BCA revealed that the protein concentration in HER-conjugated liposomes was 1.73-fold compared to control liposomes. The difference in protein content between the control and immunoliposomes is shown in Table 4.

**Table 4:** Protein content in control and immunoliposomes.

	Protein concentration (μg/mL)		
Batch	Control Liposomes	HER Liposomes	Her-to-control liposome ratio
Batch 1	46.84	81.03	1.73
Batch 2	24.42	50.08	2.05
Batch 3	46.84	95.11	2.03

#### 3.1.4 Cryogenic electron microscopy (Cryo-TEM) images

**Fig. 16:**
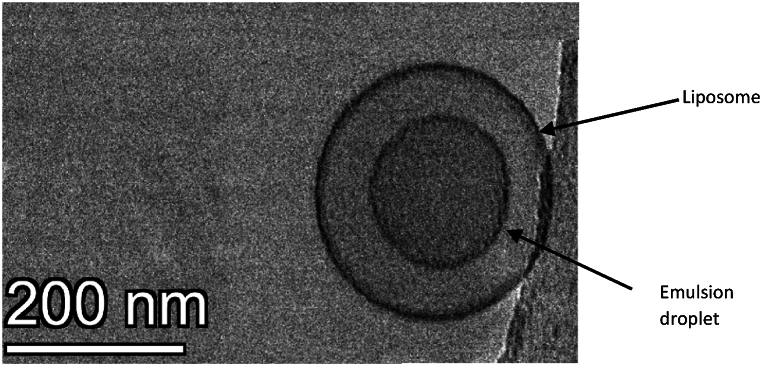
Cryo-TEM image of liposomes encapsulated with nanoemulsions*.*

Cryogenic Transmission Electron Microscopy (cryo-TEM) imaging was conducted to verify nanoemulsion encapsulation within the inner core of liposomes. The high-resolution image in Fig. 16 Serves as visual evidence, highlighting the successfully internalized nanoemulsions within the liposome. These findings are consistent and in line with the prior research studies that have showcased successful encapsulation of emulsions within liposomes [110,113], thus confirming the physical format of the eLiposomes. Furthermore, the preservation of emulsions and liposomal individual structures and the evident lack of any physical deformations affirm the suitability of liposomes.

### 3.2 Stimulation of drug release from liposomal formulations using low-frequency ultrasound (LFUS)

**Fig. 17:**
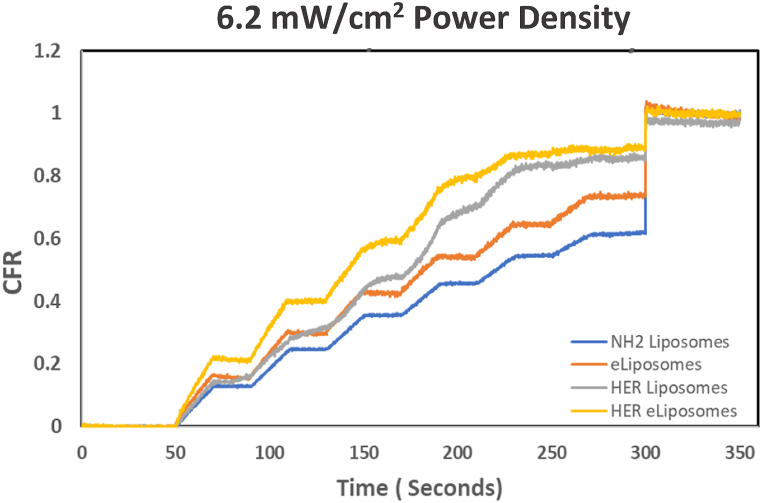
Comparison of cumulative fractional release from control liposomes, eLiposomes, HER-conjugated liposomes and HER-conjugated eLiposomes at 6.2 mW/cm^2^ power density.

**Fig. 18:**
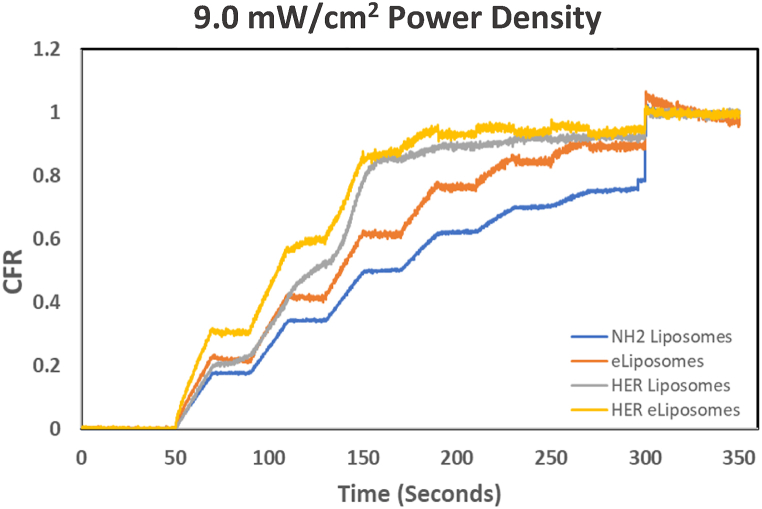
Comparison of cumulative fractional release from control liposomes, eLiposomes, HER-conjugated liposomes and HER-conjugated eLiposomes at 9.0 mW/cm^2^ power density.

**Fig. 19:**
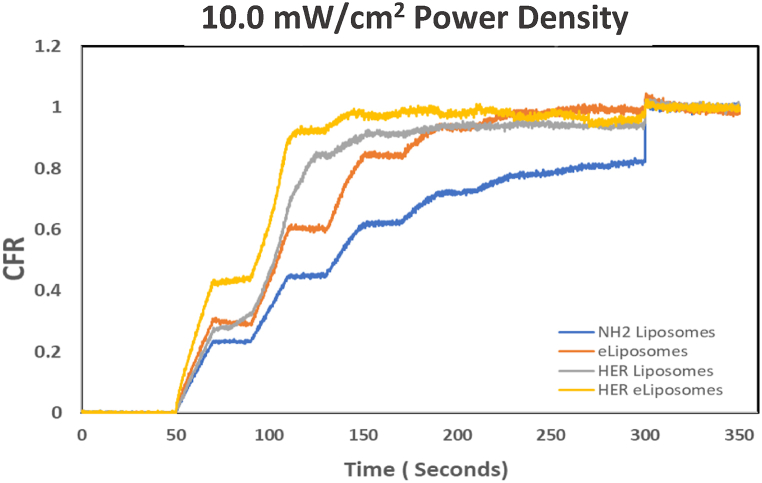
Comparison of cumulative fractional release from control liposomes, eLiposomes, HER-conjugated liposomes and HER-conjugated eLiposomes at 9.0 mW/cm^2^ power density.

Low-frequency ultrasound-controlled drug release was conducted with control liposomes, HER-conjugated liposomes, eLiposomes, and HER-conjugated eLiposomes, each comprising three replicates per batch. Three independent experiments were carried out for each liposomal formulation at different pulse power density settings. The observed pattern of release of calcein in these experiments, portrayed as cumulative release, is shown in Fig. 17, Fig. 18, Fig. 19 at three different power densities.

Initially, a baseline fluorescence, denoted by I_o_, was established for 50 s without sonication. This visualized the lack of any premature drug release and established a background fluorescence. The observation that increased fluorescence coincided with the application of ultrasound suggests that ultrasonication is the sole driving force behind the drug release. The 20-kHz sonication pulses were applied at 20-s intervals, during which each produced a notable increase in fluorescence due to the release of calcein. The change in fluorescence intensity is represented by I_t_, which has been converted in these figures to CFR according to Eqn (2). As time progressed, ultrasound-triggered drug release from liposomes reached a plateau, usually observed around the 300-s mark (5 min). At this point, surfactant Triton X-100 was introduced to lyse any remaining liposomes containing concentrated calcein, which produced a signal of the maximal release of encapsulated calcein, designated by I_100_.

Fig. 17, Fig. 18, Fig. 19 provide a comprehensive graphical representation of the dynamic changes in the drug release behavior for all liposomal formulations upon exposure to ultrasound. Furthermore, these data give insight into the relationship between US power density and the percentage of drug release at specific time intervals. The images present a positive correlation between the ultrasound power density and the CFR. A higher power density significantly increased the initial rate of release of the encapsulated drug. The drug release levels from liposomal formulations at 6.2 mW/cm^2^ during 120 s of insonation (6 pulses of 20 s each) fall short of their total release achieved using Triton-X. An apparent drug release hierarchy among the liposomal formulations can be observed, with HER-conjugated eLiposomes showcasing superior faster initial drug release, followed by HER-conjugated liposomes, eLiposomes, and finally, control liposomes (labeled NH_2_ liposomes in these figures). HER-conjugated eLiposomes show almost maximum drug release after only the 4th, 3rd, and 2nd US pulses at 6.2, 9.0, and 10.0 mW/cm^2^, respectively. The drug release potential is highly affected by encapsulation nanoemulsion droplets that are hypothesized to cause the liposomes to burst open and rapidly release the encapsulated calcein within a shorter time.

**Fig. 20:**
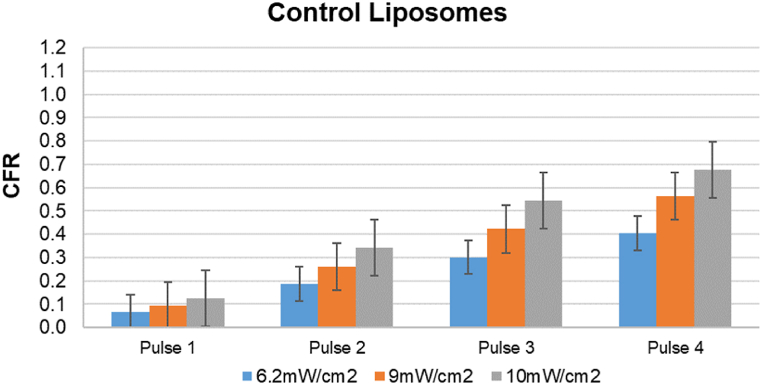
Comparison of cumulative fractional release from control liposomes after four pulses at different power densities. Error bars represent the standard deviations of repeated measurements.

**Fig. 21:**
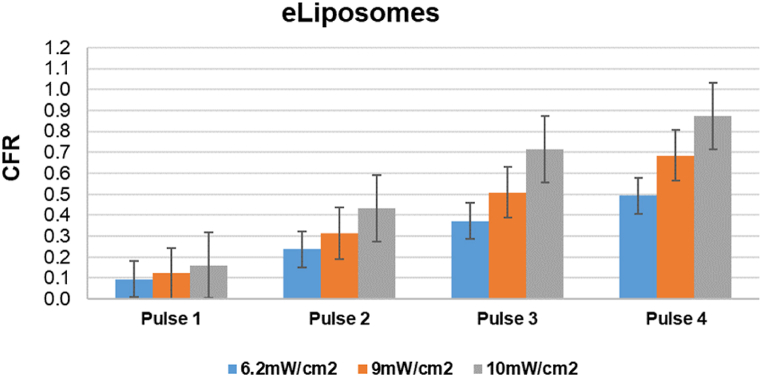
Comparison of cumulative fractional release from eLiposomes after four pulses at different power densities. Error bars represent the standard deviations of repeated measurements.

**Fig. 22:**
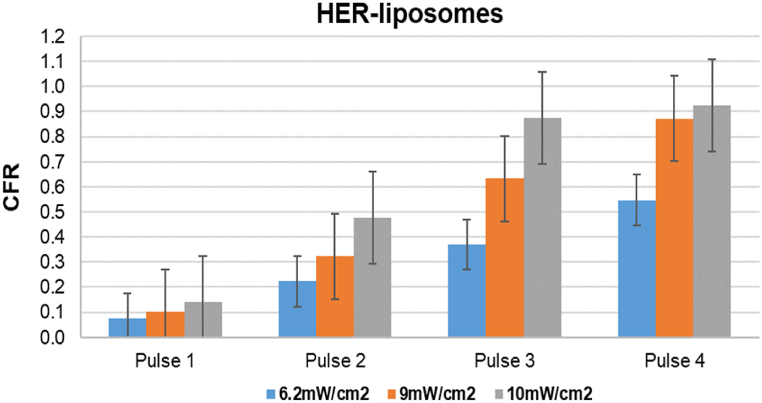
Comparison of cumulative fractional release from Herceptin-conjugated liposomes after four pulses at different power densities. Error bars represent the standard deviations of repeated measurements.

**Fig. 23:**
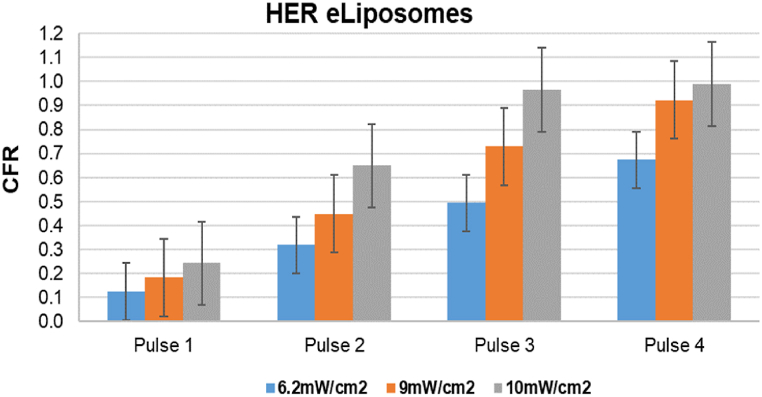
Comparison of cumulative fractional release from Herceptin-conjugated eLiposomes after four pulses at different power densities. Error bars represent the standard deviations of repeated measurements.

Fig. 20, Fig. 21, Fig. 22, Fig. 23 provide a comprehensive visual summary of the variations in the release profile of the encapsulated drug at different power densities in a bar chart format. The error bars indicate the standard deviations and highlight the minor fluctuations in drug release for each power density. The bar charts exhibit a noticeably more pronounced drug release from eLiposomes and HER-conjugated eLiposomes following the first 20-s pulse than do the control and HER-conjugated liposomes. This can be attributed to encapsulated nanoemulsions that destabilize the liposomal membrane and result in a faster drug release upon exposure to ultrasound, as discussed earlier. Moreover, it is evident that following the second pulse, both targeted liposomal formulations (i.e., HER-conjugated liposomes and eLiposomes) display an escalated drug release. This phenomenon can be attributed to the conjugated Herceptin moiety on the liposomal surface that tends to destabilize the membrane upon exposure to ultrasound.

This observation is potentially attributed to the encapsulated nanoemulsions that instantly destabilize the liposomes at the start of the sonication. On the other hand, the drug release behavior of the second US pulse demonstrates substantial statistical significance from all the liposomal formulations at different power densities.

Fig. 20, Fig. 21, Fig. 22, Fig. 23 provide a comparative insight into the effectiveness and efficacy of the formulated nanocarriers at different power densities. During the first ultrasonic pulse, eLiposomal formulations reveal a higher CFR release than other liposomes. Furthermore, the drug release achieved by HER-conjugated eLiposomes at a lower power density is comparable to that achieved by the rest of the liposomal formulations at the highest power density. Moreover, a comparative analysis of the CFR release upon exposure to a second ultrasonic pulse reveals that HER-conjugated eLiposomes demonstrate a prompt drug release rate within a short duration. This is crucial in cancer-targeted drug delivery applications where efficient and rapid drug release is prioritized.

### 3.3 Kinetic modeling

**Fig. 24:**
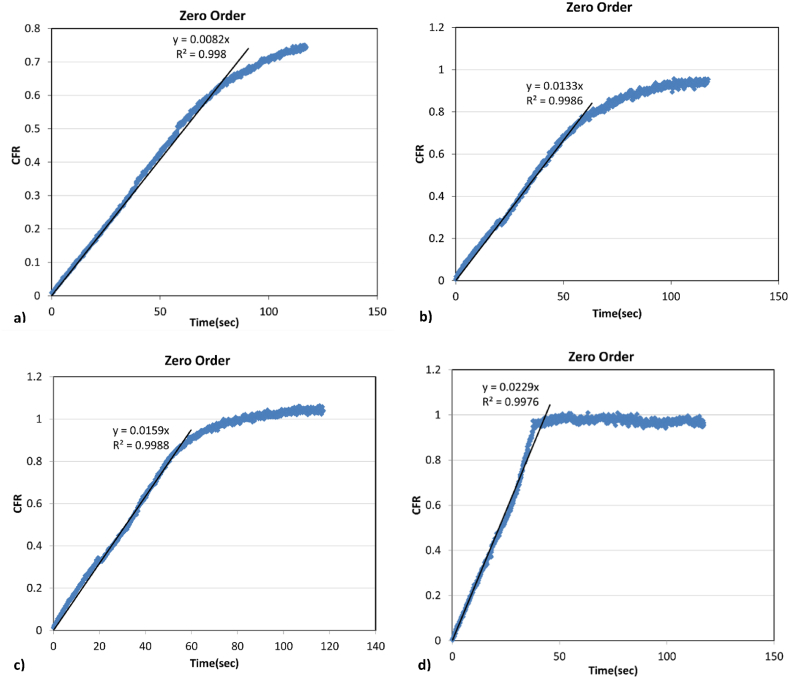
Visual representation of the zero-order release kinetics observed for a) control liposomes, b) eLiposomes, c) HER-conjugated liposomes, and HER-conjugated eLiposomes at a power density of 10.0 mW/cm.^2^.

The release data collected from experiments of control liposomes, eLiposomes, HER-conjugated liposomes and HER-conjugated eLiposomes was thoroughly analyzed by conducting release kinetics modeling. Two mathematical models, zero-order and first-order release kinetics, are employed to study the release behavior of the encapsulated drug, and the goodness of fit for both models is evaluated using the R^2^ value. Fig. 24 visually represents the release kinetics from different liposomal formulations at a power density of 10.0 mW/cm^2^. In this figure, the data from times at which no ultrasound was applied has been removed, effectively “sliding” the data to the left so that only data during insonation are shown. This connects the insonated data together in a single plot. It can be observed that the behavior of all liposomal formulations, when subjected to ultrasonic triggers at different power densities, caused the drug to be released at a nearly constant rate over time, regardless of the concentration during each pulse individually. All liposomal formulations (control liposomes, eLiposomes, HER-conjugate liposomes and HER-conjugated eLiposomes) were best fit by zero-order release kinetics over an initial time-course, followed by a transition to first-order kinetics at later times. The time of transition from zero-order to first-order increased in the sequence of control, eLiposomes, HER-liposomes, and HER-eLiposomes. This is the same sequence as the increase in the initial zero-order release rate constant, given as the slopes shown in Fig. 24. Understanding the liposomal release and delivery behavior helps in designing effective liposomes for targeted cancer delivery.

### 3.4 Discussion

Targeted drug delivery offers a promising strategy to deliver therapeutic agents directly to the diseased site, thus enhancing the treatment efficacy while minimizing systemic drug interaction with healthy cells and reducing potential side effects. Targeted nanocarriers offer diverse material properties that enable personalized treatment by tailoring drug delivery systems. Liposomes are among the most widely and successfully used nanocarriers due to their remarkable attributes associated with exceptional biodegradability, biocompatibility, and ability to encapsulate universal drugs, including hydrophilic and hydrophobic compounds.

Liposomes provide a highly adaptable platform for surface modification, facilitating the attachment of various moieties to their outer surfaces. This empowers researchers to target distinct receptors overexpressed on cancer cell surfaces. Liposomes used in this study were coated with polyethylene glycol (PEG) that will protect the liposomes against clearance from the bloodstream, thereby extending liposomal blood circulation time and allowing extravasation through the tumor leaky vasculature. Moreover, antibodies play a compelling role in targeting strategies and can be conjugated to liposomal surfaces to form immunoliposomes. These immunoliposomes can effectively target overexpressed receptors like HER2 receptors on the surface of breast cancer cells, thus enhancing the treatment efficiency and reducing off-target effects associated with the chemotherapeutic drugs.

Our ongoing investigation studied the impact of encapsulating PFC_5_ nanoemulsions and conjugating a monoclonal antibody for HER2 receptors, known as Trastuzumab (Herceptin), onto pegylated liposomes of small size (<200 nm). The physical and chemical properties of all liposomal formulations were compared and analyzed. Cryo-TEM images provided evidence supporting the proposed construct of nanoemulsions encapsulated within liposomes. Release of encapsulated calcein from formulated nanocarriers was triggered and controlled using low-frequency ultrasound (LFUS) at 20 kHz in a pulse mode. The drug release escalated significantly with the increase in the power density. Furthermore, pulsed-mode ultrasound demonstrated that ultrasound was the sole driving force behind the drug release, with no substantial release observed during the inactive period between the pulses.

The fastest release rates were demonstrated by HER-conjugated eLiposomes at various power densities, thus revealing their superior performance compared to other liposomal formulations. Each successive pulse achieved an increased drug release at higher power densities. Furthermore, the HER-eLiposomes showed sustained zero-order release kinetics and, achieved almost full release by the end of the second 20-s pulse at 10.0 mW/cm^2^. Furthermore, it is worth noting that HER-conjugated eLiposomes released as much drug at the lowest power density as released by control liposomes at the highest power density used in this study. These observations imply that both encapsulation of nanoemulsions within liposomes and conjugation of a heavy receptor target to the liposomal surface significantly help modulate the drug release behavior. Yet remarkably, the HER-eLiposomes remain intact and immediately stop release as soon as the ultrasound exposure is stopped. Using lower power density for cancer-targeted treatment helps reduce heat induction or tissue damage caused by high power intensities, thus providing controlled drug release in space and time and increasing efficacy and treatment effectiveness.

The calcein release from liposomes was fit to zero-order and first-order kinetic models, and this drug delivery system most closely followed zero-order kinetics for HER-eLiposomes. However, the release from control liposomes started with only a short segment of zero-order kinetics followed by first-order release kinetics. The other types of liposome formulations fell between these extremes. Such data shows that the HER-eLiposomes are much more fragile toward ultrasonic disruption than control liposomes, and the eLiposomes and HER-liposomes lie somewhere in between.

These data present a complex disruption model caused by the energy of ultrasound and cavitation events. Herein we propose a simplified model wherein if energy exceeds a threshold of fragility, the liposome breaks. Conjugating antibodies to the exterior of liposomes appears to make the liposome more fragile. Placing emulsions inside the liposome appears to contribute to more powerful stress in disrupting the liposome and releasing the calcein. Both fragility and cavitation potential have statistical contributions. If all cavitation events are more powerful than fragility, then all liposomes are disrupted at the rate that cavitation happens, but not proportional to the concentration of intact liposomes; this would lead to zero-order kinetics. At higher ultrasound intensities, more cavitation occurs, and the zero-order rate constant is larger, producing faster drug release. This appears to be the case with HER-eLiposomes. Fragility and emulsions contribute significantly to zero-order rupture.

For more robust liposomes, those lacking HER2-antibodies and/or lacking emulsions, there appears to be a distribution of fragilities in these populations. Our model proposes that initially, the fraction of that distribution that is susceptible to ultrasound damage at a given intensity is quickly disrupted with zero-order kinetics, while the rest of the population is disrupted with first-order kinetics. Further development of this qualitative model is left for future studies.

Whatever the molecular mechanisms underlying release from these types of liposomes, the results obtained from this study highlight the potential of nanoemulsions along with targeting moieties and low-frequency ultrasound (LFUS) to trigger, control, and enhance the release of therapeutic drugs from liposomes.

## 4 Conclusion

In this study, we successfully synthesized nanoemulsions and encapsulated them within Herceptin-conjugated liposomes. Moreover, we investigated the impact of LFUS at different power densities to induce controlled drug release from various liposome formulations. Adding emulsion droplets into the liposome increased the fragility of the construct toward drug release, as did placing Herceptin antibodies on the liposome surface, with the most ultrasonically-labile constructs having both. Very rapid zero-order release resulted from low-frequency insonation. The combination of nanoemulsions with HER2 conjugation produced a powerful combination that quickly released calcein from the liposomes.

As for clinical application, the presence of HER2 receptors on the surface of breast cancer cells provides a promising approach for utilizing HER2-targeted liposomes that facilitate direct delivery of the therapeutic agents to the cancer cells and enhance treatment efficacy. These findings provide valuable information for further research and development of future in-vitro and in-vivo studies that aim to exploit the overexpression of Herceptin and rapidly release the drug upon ultrasonication. This research is promising in improving the prognosis of cancer patients and rendering chemotherapy more humane by minimizing the side effects associated with conventional chemotherapy, ultimately enhancing patient well-being and quality of life.

## Data availability

The datasets used and/or analyzed during the current study are available from the corresponding author on reasonable request.

## Funding

This research study was funded by the Dana Gas Endowed Chair for Chemical Engineering, the 10.13039/501100002724American University of Sharjah Faculty Research Grants (FRG20-L-E48, FRG22-C-E08), 10.13039/100012002Sheikh Hamdan Award for Medical Sciences (MRG/18/2020), and Friends of Cancer Patients (FoCP).

## CRediT authorship contribution statement

**Mah Noor Zafar:** Writing – original draft, Visualization, Validation, Methodology, Investigation, Formal analysis, Data curation, Conceptualization. **William G. Pitt:** Writing – review & editing, Validation, Supervision, Conceptualization. **Ghaleb A. Husseini:** Writing – review & editing, Supervision, Resources, Project administration, Methodology, Investigation, Funding acquisition, Conceptualization.

## Declaration of competing interest

The authors declare that they have no known competing financial interests or personal relationships that could have appeared to influence the work reported in this paper.
